# GOREA: Unbiased Interpretation of Functional Enrichment

**DOI:** 10.1016/j.mocell.2025.100283

**Published:** 2025-09-24

**Authors:** Hojin Lee, Young-In Park, Ina Jeon, Dawon Kang, Harim Chun, Jungmin Choi

**Affiliations:** Department of Biomedical Sciences, College of Medicine, Korea University, Seoul 02841, Republic of Korea

**Keywords:** Gene ontology biological process, Gene set enrichment analysis, GOREA, Over-representation analysis, simplifyEnrichment

## Abstract

Functional enrichment analysis is essential for extracting biological meaning from gene expression data. Gene set enrichment analysis (GSEA) and over-representation analysis (ORA) are widely used approaches for this purpose. However, interpreting the large number of enriched gene ontology biological process (GOBP) terms remains challenging. Existing tools such as simplifyEnrichment often yield overly general and fragmented keywords, and they do not effectively utilize quantitative metrics such as normalized enrichment scores (NES) or gene overlap proportions, thereby limiting biological interpretation and prioritization. To address these issues, we developed GOREA, an improved tool for summarizing GOBP terms. GOREA improves upon simplifyEnrichment by integrating binary cut and hierarchical clustering, incorporating GOBP term hierarchy to define representative terms, and ranking clusters based on NES or gene overlap proportions. Using ComplexHeatmap R package, GOREA visualizes results as a heatmap accompanied by a panel of broad GOBP terms and representative terms for each cluster, providing both general and specific biological insights. Compared to simplifyEnrichment, GOREA yields more specific and interpretable clusters while significantly reducing computational time. GOREA effectively identified distinct biological processes in immune-related data and revealed substantial overlap between GOBP terms and cancer hallmark gene sets, demonstrating its applicability across diverse biological contexts. These findings suggest that GOREA provides a substantial improvement over existing approaches and offers a scalable and efficient framework for GSEA and ORA across diverse biological contexts.

## INTRODUCTION

To extract biological meaning from gene expression data, functional enrichment analysis is essential. The most well-established methods for this purpose are gene set enrichment analysis (GSEA) and over-representation analysis (ORA) (Kuleshov et al., 2016, Subramanian et al., 2005). Gene ontology (GO), a structured representation of gene function, supports data interpretation across biology (Ashburner et al., 2000). GO consists of 3 main categories: (1) biological process (BP), (2) molecular function (MF), and (3) cellular component (CC) (Ashburner et al., 2000). Among these, BP is widely used to describe biological context. However, because these results often contain numerous GO terms, researchers typically report only the top 10 terms (Fig. S1A-F) (Anerillas et al., 2023, Khan et al., 2019, Yoshida et al., 2022). This practice can limit a comprehensive understanding of the data and may lead to missed discovery opportunities.

## MAIN BODY

### Challenges in simplifyEnrichment

**Fig. 1:**
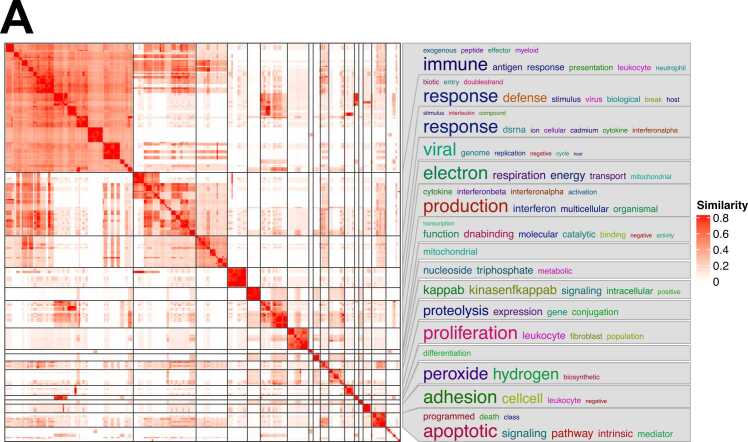
Heatmap showing the result of 277 gene ontology biological pathway (GOBP) terms using binary cut.

simplifyEnrichment was developed to address difficulties in interpreting enriched terms (Gu and Hubschmann, 2023). The clustering results, obtained using the binary cut method, revealed both large and small clusters of functionally enriched terms (Figs. 1A and S2A). Fragmented keywords representing the clusters were displayed in the right panel of Figure 1A. However, the biological interpretation of these clusters remains difficult. For example, simplifyEnrichment identified general terms such as “regulation,” “transcription,” “proteolysis,” “expression,” “dnabinding,” “gene,” and “conjugation” (Fig. 1A), which do not sufficiently capture the specific biological context of each cluster. Moreover, simplifyEnrichment does not report quantitative measures such as the normalized enrichment score (NES) or the proportion of overlapping genes relative to the total number of genes in each gene ontology biological process (GOBP) term, making it challenging to prioritize clusters (Fig. 1A).

#### Overview of GOREA: Overcoming the Limitations of simplifyEnrichment

**Fig. 2:**
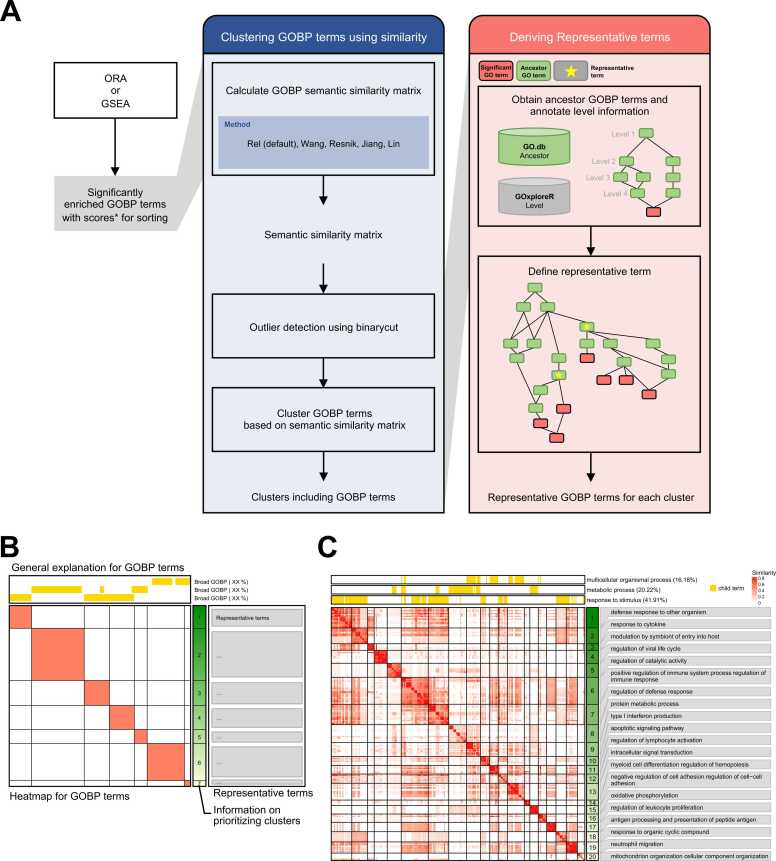
Algorithm and the result of GOREA. (A) Schematic representation for GOREA, indicating major steps of the tool. Significant gene ontology (GO) ID can be summarized by blue box and red box. The blue box is a step for clustering, and the red box is a step for defining representative terms for each cluster. *Score; normalized enrichment score (NES) from gene set enrichment analysis (GSEA) and the proportion of overlapping genes from ORA can be utilized as input data. When using NES, the sign of the GO items must be considered by using GO terms with positive or negative NES in each GOREA analysis. (B) Simplified results for GOREA. (C) Heatmap showing the result of 277 GOBP terms using combined clustering.

We introduce GOREA, an acronym derived from GO, the R programming language (R), ORA, and GSEA. GOREA requires input of significant GOBP terms with either overlap proportion or NES (Fig. 2A). Clustering is performed using a combined method that integrates binary cut and hierarchical clustering (see Supplementary information, Figs. 2A and S2A-D). To define representative terms for each cluster, the algorithm incorporates information on ancestor terms and GOBP term levels from GOxplore (Manjang et al., 2020), R package. Specifically, the process involves (1) identifying the highest-level common ancestor term encompassing a subset of the input GOBP terms, and (2) repeating it for the remaining terms not covered by the representative term identified in step (1) (Fig. 2A). Using the R package ComplexHeatmap (Gu et al., 2016), clusters are visualized as a heatmap with representative terms displayed on the right, sorted by average gene overlap or NES (Fig. 2B). To support a broader biological overview, a panel of broad GOBP terms is positioned above the heatmap, with each term labeled by the percentage of included child GOBP terms (see Supplementary information, Figs. 2B and S2E) (Manjang et al., 2020).

In a comparative analysis, GOREA identified distinct immune-related clusters such as “defense response to other organism,” “response to cytokine,” and “antigen processing and presentation of peptide antigen” (Fig. 2C). In contrast, simplifyEnrichment grouped these immune-related terms into a single, broad cluster (Fig. 1A), highlighting GOREA’s ability to capture more specific biological insights. Moreover, terms such as “oxidative phosphorylation” were captured only by GOREA (Fig. 2C). Next, we compared the representative terms in the right panel between GOREA and simplifyEnrichment (Figs. 1A and 2C). For example, in cluster 4, GOREA identified “regulation of viral life cycle,” whereas simplifyEnrichment produced “viral,” “genome,” “replication,” “negative,” “cycle,” “regulation,” and “host.” Overall, this example demonstrates that GOREA yields more human-readable representative terms and enables biological interpretation than simplifyEnrichment.

#### Performance and Efficiency Evaluation of GOREA

**Fig. 3:**
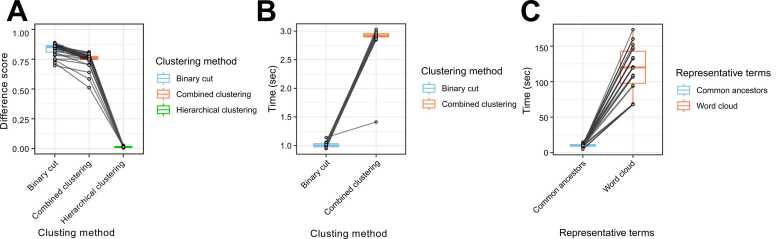
Performance and efficiency of GOREA. (A) Box plot indicating difference score of 3 clustering methods. (B) Box plot indicating time consumption in the clustering steps from simplifyEnrichment and GOREA. (C) Box plot indicating time consumption when obtaining representative terms in simplifyEnrichment and GOREA.

To evaluate the performance of the clustering methods, we compared their difference scores, which quantify how well clusters are separated. The combined clustering approach implemented in GOREA demonstrated significantly lower difference scores than the binary cut method (Wilcoxon signed-rank test, *P* = 3.47e−07), indicating improved clustering precision. However, its difference scores were significantly higher than those of the hierarchical clustering method (Wilcoxon signed-rank test, *P* < 2.2e−16, Fig. 3A). Regarding computational efficiency, the binary cut method required an average of 1.01 seconds for the clustering step, while the combined clustering method took approximately 2.88 seconds (Fig. 3B). For the step involving presentation of representative terms, the method using common ancestor—as applied in GOREA—had an average processing time of 9.98 seconds, whereas the word cloud-based approach employed by simplifyEnrichment required approximately 118 seconds (Fig. 3C). This demonstrates a substantial efficiency gain with the GOREA approach. By reducing overall processing time, GOREA enables researchers to perform such iterative optimization more efficiently, thus accelerating the biological interpretation workflow.

#### Application of GOREA in Cancer Biology Interpretation

We first applied GOREA to immune-related data and found that it successfully captured diverse BPs with enhanced interpretability (Fig. 2C). To further assess GOREA’s utility in a more complex biological context, we explored its application in cancer biology. We analyzed cancer data using GSEA with the Hallmark database, which is a well-established approach for extracting biological meaning from cancer-related data (Liberzon et al., 2015). Interestingly, the results obtained from GSEA using the Hallmark gene sets could not be reproduced when the GOBP database was used (Fig. S3A and S3B). While Hallmark and GOBP shared same keywords, the overall proportion of overlapping genes was low, indicating fundamental differences between the Hallmark and GOBP terms (see Supplementary information, Fig. S3C and S3D). To further explore the relationship between GOBP terms and cancer hallmark gene sets, we calculated the proportion of overlapping genes between these 2 resources (Otília Menyhart and Balázs, 2025). This analysis identified 132 GOBP terms that were included within the Hallmark gene sets, suggesting that GOREA effectively captures cancer-related BPs (see Supplementary information, Fig. 4A). These findings support the use of GOREA for interpreting ORA or GSEA results based on GOBP terms in cancer research.

**Fig. 4:**
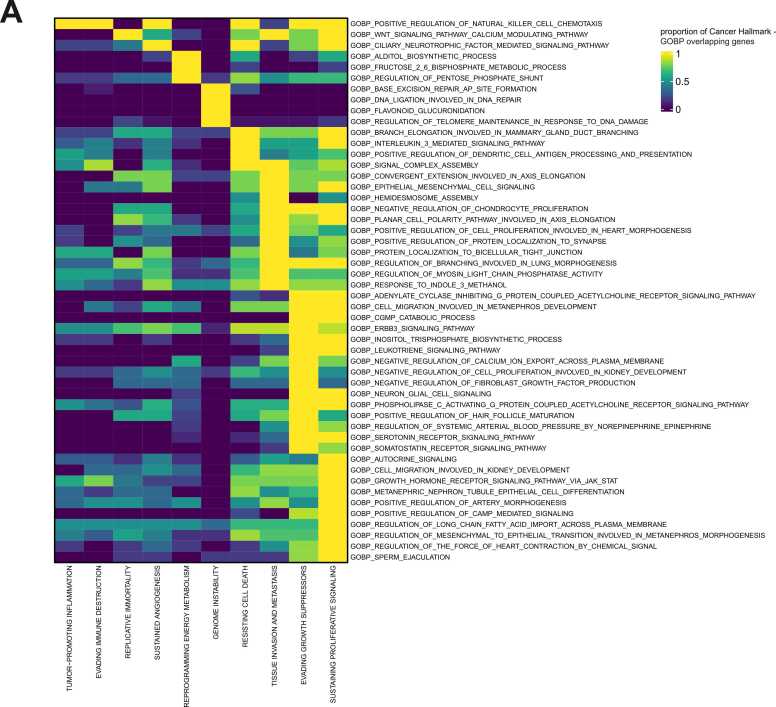
Heatmap showing proportion of overlapping genes between cancer hallmark and the latest GOBP (v24.1) from MsigDB. The red GOBP terms on the x-axis are GOBP terms found to be similar when compared to hallmark data from MsigDB. For visualization, a random subset of 50 GOBP terms was selected from the total of 132 GOBP term.

#### Limitations

GOREA requires an ontology with an explicit hierarchical structure. Accordingly, it is designed for GO categories (BP, CC, MF) and does not directly operate on nonhierarchical gene-set collections such as MSigDB Hallmark or KEGG gene sets. Users seeking to analyze these resources may run them in parallel with GO-based analysis; GOREA should then be applied to the GO results.

## CONCLUDING REMARKS

In conclusion, GOREA introduces novel clustering methods, a strategy for defining representative terms, and a panel for broad GOBP terms, providing researchers an intuitive and effective way to extract biological insights from their data. By sorting clusters based on NES or the proportion of overlapping genes, GOREA also enables better prioritization of biologically relevant clusters. In terms of computational efficiency, GOREA outperformed simplifyEnrichment. Importantly, this approach is applicable to both cancer and noncancer datasets, facilitating comprehensive understanding of biological contexts. The GOREA source code is freely available as an R script at https://github.com/KuChoiLab/GOREA.

## Funding and Support

This work was supported by the National Research Foundation of Korea (RS-2023-00212238) and the Institute of Information & Communications Technology Planning & Evaluation-ICT Challenge and Advanced Network of HRD grant (IITP-2024-RS-2024-00438263).

## CRediT authorship contribution statement

**Young-in Park:** Visualization, Formal analysis. **Dawon Kang:** Visualization, Formal analysis. **Ina Jeon:** Visualization, Formal analysis. **Jungmin Choi:** Writing – review & editing, Supervision, Conceptualization. **Harim Chun:** Visualization, Data curation. **Hojin Lee:** Writing – original draft, Formal analysis.

## Declaration of Competing Interest

The authors declare that there are no conflicts of interest.

## Appendix A Supplementary material

.

Legned for Supplementary Figures

## Footnotes

Appendix A Supplemental material associated with this article can be found online at doi:10.1016/j.mocell.2025.100283.
