# Supplementary material for: GOREA: Unbiased Interpretation of Functional Enrichment
Source: Mol Cells. 2025 Sep 24;48(11):100283. doi: 10.1016/j.mocell.2025.100283 (PMC12552962; doi:10.1016/j.mocell.2025.100283)
Supplement: Supplementary file 1 — Supplementary Information [file mmc1.pdf]

Supplementary information for
GOREA: Unbiased Interpretation of Functional Enrichment

Hojin Lee[1], Young-in Park[1], Ina Jeon[1], Dawon Kang[1], Harim Chun[1], and Jungmin Choi[1*]

[1]Department of Biomedical Sciences, College of Medicine, Korea University, Seoul 02841, Republic of Korea

[*]Corresponding author.

Development of combined methods

In terms of clustering, a hierarchical clustering-based combined clustering approach is applied in a similarity matrix generated by semantic similarity. We identified the strengths of both clustering methods: (1) Binary cut (Gu and Hubschmann, 2023) effectively detected small clusters, (2) Hierarchical clustering allowed for intuitive control over the number of clusters; therefore, we developed combined clustering method by taking the two strengths. First, to detect small clusters, binary cut clustering is performed. Then, hierarchical clustering is applied, and the small clusters are removed from the final hierarchical clustering results (Fig. 2A).

Comparison among clustering methods

A comparison between binary cut and hierarchical clustering with the same number of clusters revealed that hierarchical clustering failed to detect small clusters and instead produced more uniformly sized clusters than binary cut (Fig. S2A). To further investigate the characteristics of the binary cut clustering method, 500 random GOBP terms were sampled 30 times. Subsequently, binary cut clustering was applied to the 30 GOBP sets (Fig. S2B). When the cutoff for binary cut was gradually decreased from the default value of 0.85 in steps of 0.01, the number of clusters did not increase gradually with the decreasing cutoff. Instead, it resulted in an unpredictable number of clusters (Fig. S2B). This made it difficult for researchers to regulate the number of clusters when aiming to identify a specific level of biological meaning from significant GO terms. Binary cut well-defined small clusters, which increased with decreasing cutoff. However, large clusters remained unchanged, even when the number of clusters was doubled (Fig. S2B). For hierarchical clustering, an intuitive number of clusters can be obtained when increasing the number of clusters using the 'cuttree' function from stats R package (Fig. S2C). Additionally, hierarchical clustering showed a gradual decrease in the size of the largest clusters as the total number of clusters increased (Fig. S2C). For combined clustering, in each simulation, small clusters were added, and the total number of clusters was controlled according to a predefined value, k (Fig. S2D).

Defining broad GOBP terms

A panel of broad GOBP terms was applied in our tool. To suggest a general description of input GOBP terms, broad GOBP terms were defined. Among 125 GOBP terms with level 1 and 2 based on GOxplore (Manjang et al., 2020) R package, after which 3 GOBP terms were removed, because the terms were identified as outliers, including a large number of child terms using Grubb's test through 'grubbs.test' function from outliers R package (Fig. S2E). Broad GOBP terms, including input GOBP terms as their child terms, were clustered. For each cluster, the

broad GOBP term that includes the highest number of child GOBP terms was selected and displayed, along with the percentage of significant terms it covers (Fig. 2B).

Comparison between GOBP and Hallmark

To identify the underlying cause of this discrepancy, we conducted a comparative analysis between the Hallmark and GOBP databases (Fig. S3C and S3D). First, we identified GOBP terms sharing the same or similar keywords with Hallmark. Next, we calculated the proportion of overlapping genes between the identified GOBP terms and the Hallmark gene sets. To account for differences across databases of GOBP, we analyzed the latest versions of GOBP from MsigDB (https://www.gsea-msigdb.org/gsea/msigdb) and Gene Ontology (https://geneontology.org/docs/download-ontology/) (Liberzon et al., 2015) (Ashburner et al., 2000).

Comparison between GOBP and cancer hallmark

Additionally, the proportion of overlapping genes between the GOBP terms and cancer hallmark gene sets (https://cancerhallmarks.com/) was calculated to explore cancer hallmark gene sets further (Otília Menyhart, 2025). After that, GOBP terms that include all genes from at least one cancer hallmark gene set remained (Fig. 4A).

References

Ashburner, M., Ball, C.A., Blake, J.A., Botstein, D., Butler, H., Cherry, J.M., Davis, A.P., Dolinski, K., Dwight, S.S., Eppig, J.T., et al. (2000). Gene ontology: tool for the unification of biology. The Gene Ontology Consortium. Nat Genet 25, 25-29.

Gu, Z. and Hubschmann, D. (2023). simplifyEnrichment: A Bioconductor Package for Clustering and Visualizing Functional Enrichment Results. Genomics Proteomics Bioinformatics 21, 190-202.

Liberzon, A., Birger, C., Thorvaldsdottir, H., Ghandi, M., Mesirov, J.P., and Tamayo, P. (2015). The Molecular Signatures Database (MSigDB) hallmark gene set collection. Cell Syst 1, 417-425.

Manjang, K., Tripathi, S., Yli-Harja, O., Dehmer, M., and Emmert-Streib, F. (2020). Graph-based exploitation of gene ontology using GOxploreR for scrutinizing biological significance. Sci Rep-Uk 10.

Otília Menyhart, W.J.K., Balázs Győrffy (2025). A gene set enrichment analysis for cancer hallmarks. Journal of Pharmaceutical Analysis 15.
