# Supplementary material for: GOREA: Unbiased Interpretation of Functional Enrichment
Source: Mol Cells. 2025 Sep 24;48(11):100283. doi: 10.1016/j.mocell.2025.100283 (PMC12552962; doi:10.1016/j.mocell.2025.100283)
Supplement: Supplementary file 2 — Supplementary Figures [file mmc2.pdf]

Fig. S1

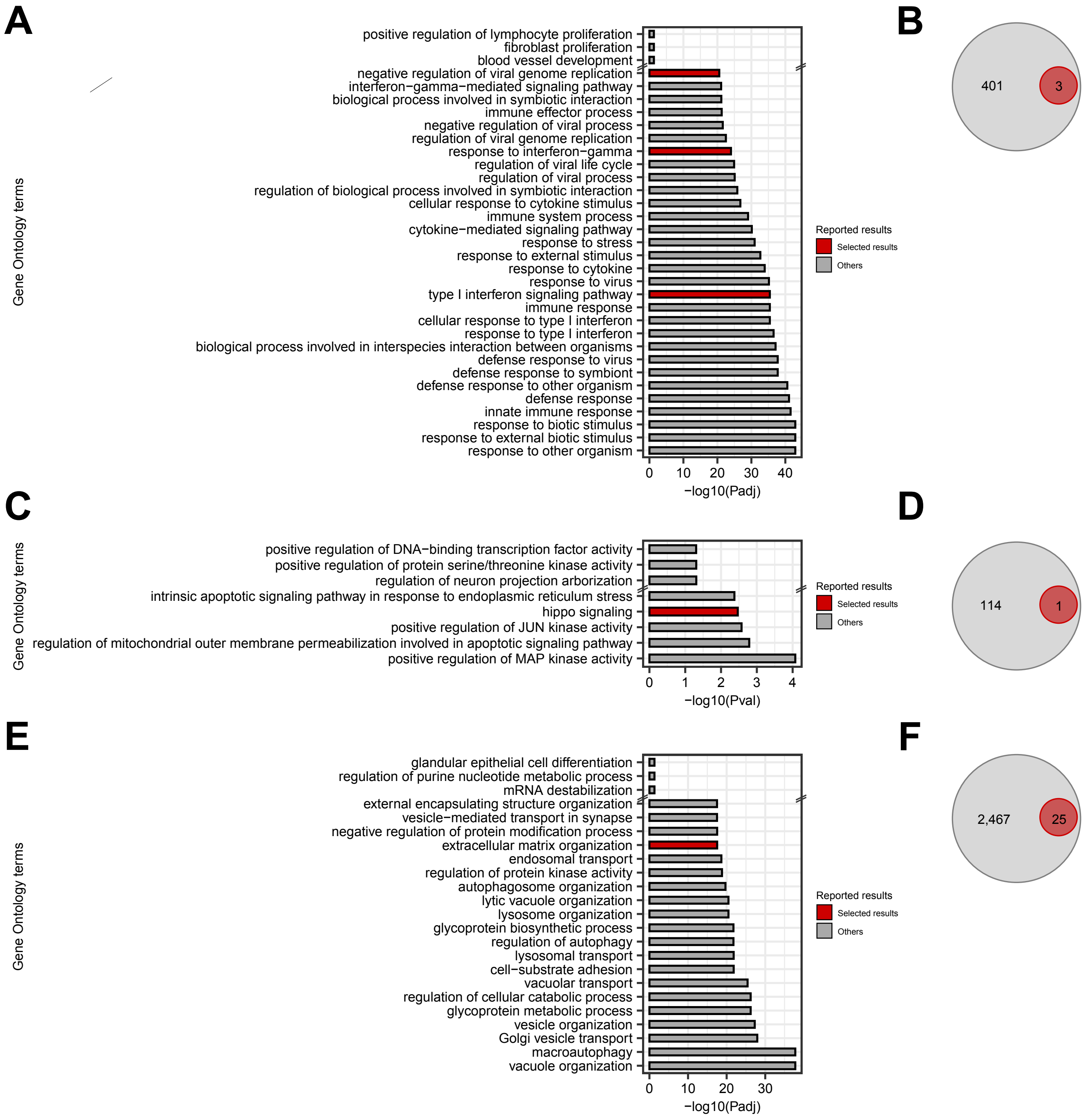
A
positive regulation of lymphocyte proliferation
fibroblast proliferation
blood vessel development
negative regulation of viral genome replication
interferon−gamma−mediated signaling pathway
biological process involved in symbiotic interaction
immune effector process
negative regulation of viral process
regulation of viral genome replication
response to interferon−gamma
regulation of viral life cycle
regulation of viral process
regulation of biological process involved in symbiotic interaction
cellular response to cytokine stimulus
immune system process
cytokine−mediated signaling pathway
response to stress
response to external stimulus
response to cytokine
response to virus
type I interferon signaling pathway
immune response
cellular response to type I interferon
response to type I interferon
biological process involved in interspecies interaction between organisms
defense response to virus
defense response to symbiont
defense response to other organism
defense response
innate immune response
response to biotic stimulus
response to external biotic stimulus
response to other organism
Gene Ontology terms
0 10 20 30 40
−log10(Padj)
Reported results
Selected results
Others
B
401
3
C
positive regulation of DNA−binding transcription factor activity
positive regulation of protein serine/threonine kinase activity
regulation of neuron projection arborization
intrinsic apoptotic signaling pathway in response to endoplasmic reticulum stress
hippo signaling
positive regulation of JUN kinase activity
regulation of mitochondrial outer membrane permeabilization involved in apoptotic signaling pathway
positive regulation of MAP kinase activity
Gene Ontology terms
0 1 2 3 4
−log10(Pval)
Reported results
Selected results
Others
D
114
1
E
glandular epithelial cell differentiation
regulation of purine nucleotide metabolic process
mRNA destabilization
external encapsulating structure organization
vesicle−mediated transport in synapse
negative regulation of protein modification process
extracellular matrix organization
endosomal transport
regulation of protein kinase activity
autophagosome organization
lytic vacuole organization
lysosome organization
glycoprotein biosynthetic process
regulation of autophagy
lysosomal transport
cell−substrate adhesion
vacuolar transport
regulation of cellular catabolic process
glycoprotein metabolic process
vesicle organization
Golgi vesicle transport
macroautophagy
vacuole organization
Gene Ontology terms
0 10 20 30
−log10(Padj)
Reported results
Selected results
Others
F
2,467
25

Fig. S2

A

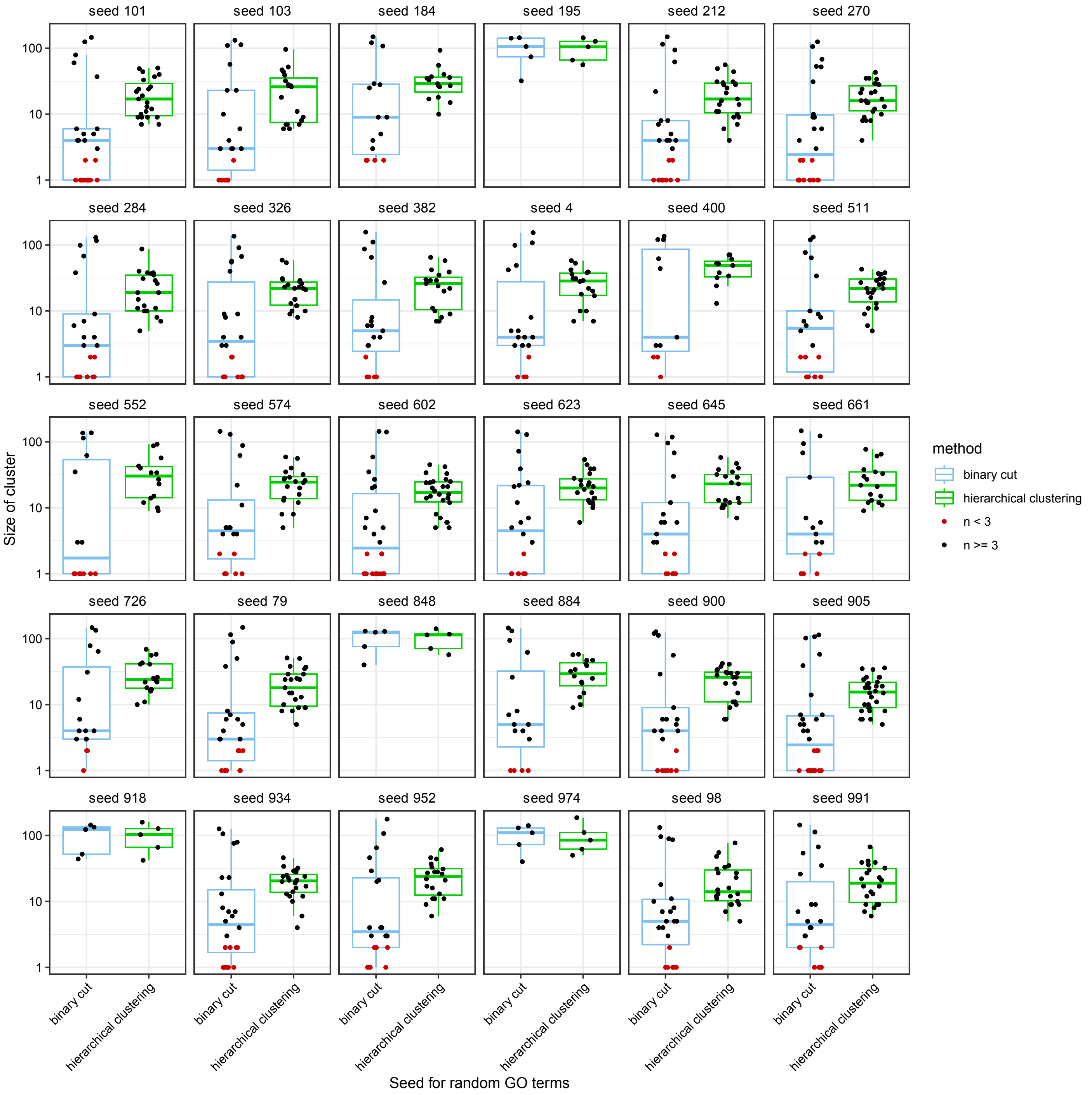

seed 101
seed 103
seed 184
seed 195
seed 212
seed 270
seed 284
seed 326
seed 382
seed 4
seed 400
seed 511
seed 552
seed 574
seed 602
seed 623
seed 645
seed 661
seed 726
seed 79
seed 848
seed 884
seed 900
seed 905
seed 918
seed 934
seed 952
seed 974
seed 98
seed 991
100
10
1
Size of cluster
binary cut
hierarchical clustering
Seed for random GO terms
method
binary cut
hierarchical clustering
n < 3
n >= 3

B

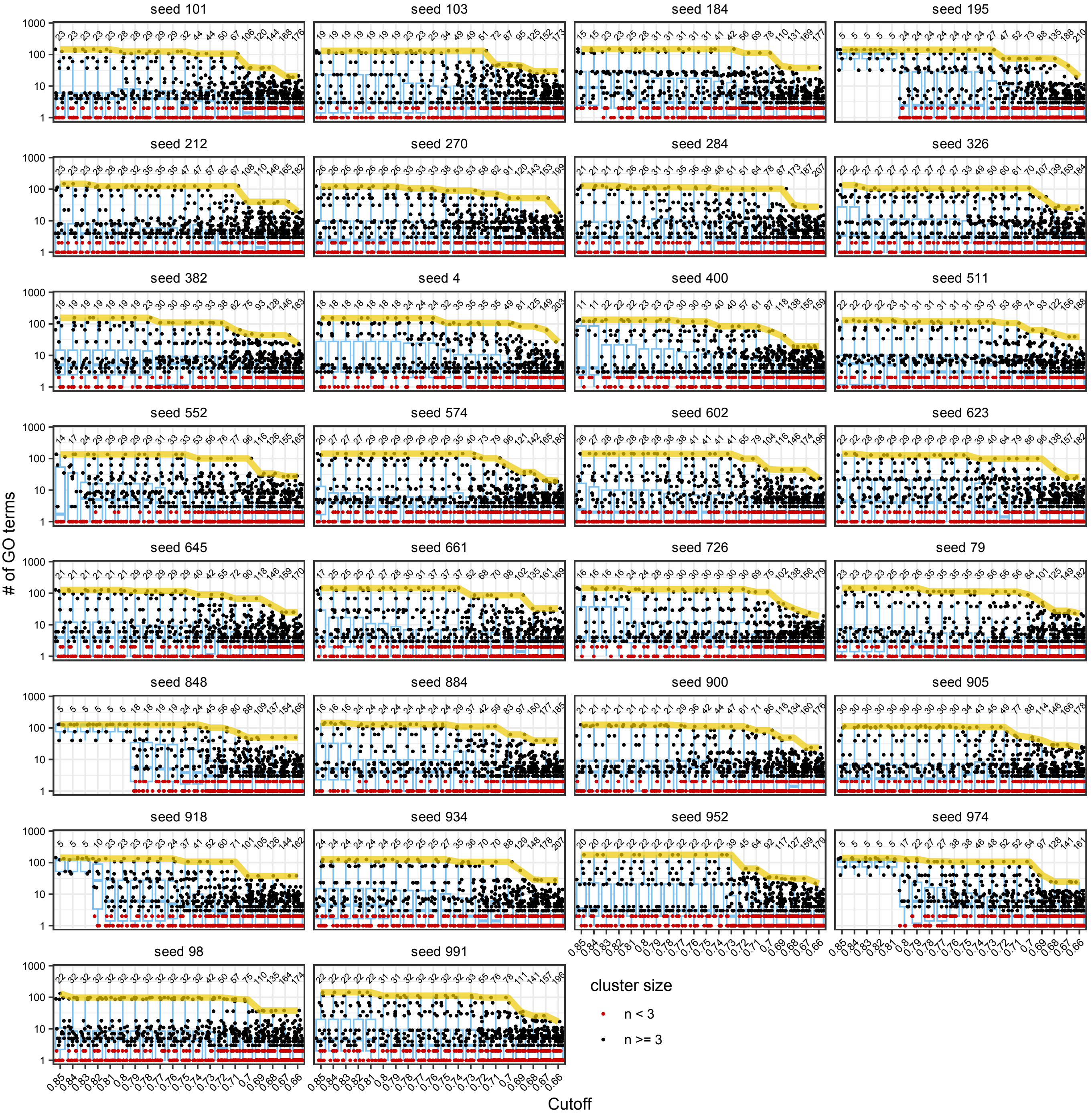
seed 101
seed 103
seed 184
seed 195
seed 212
seed 270
seed 284
seed 326
seed 382
seed 4
seed 400
seed 511
seed 552
seed 574
seed 602
seed 623
seed 645
seed 661
seed 726
seed 79
seed 848
seed 884
seed 900
seed 905
seed 918
seed 934
seed 952
seed 974
seed 98
seed 991
# of GO terms
Cutoff
cluster size
n < 3
n >= 3

C

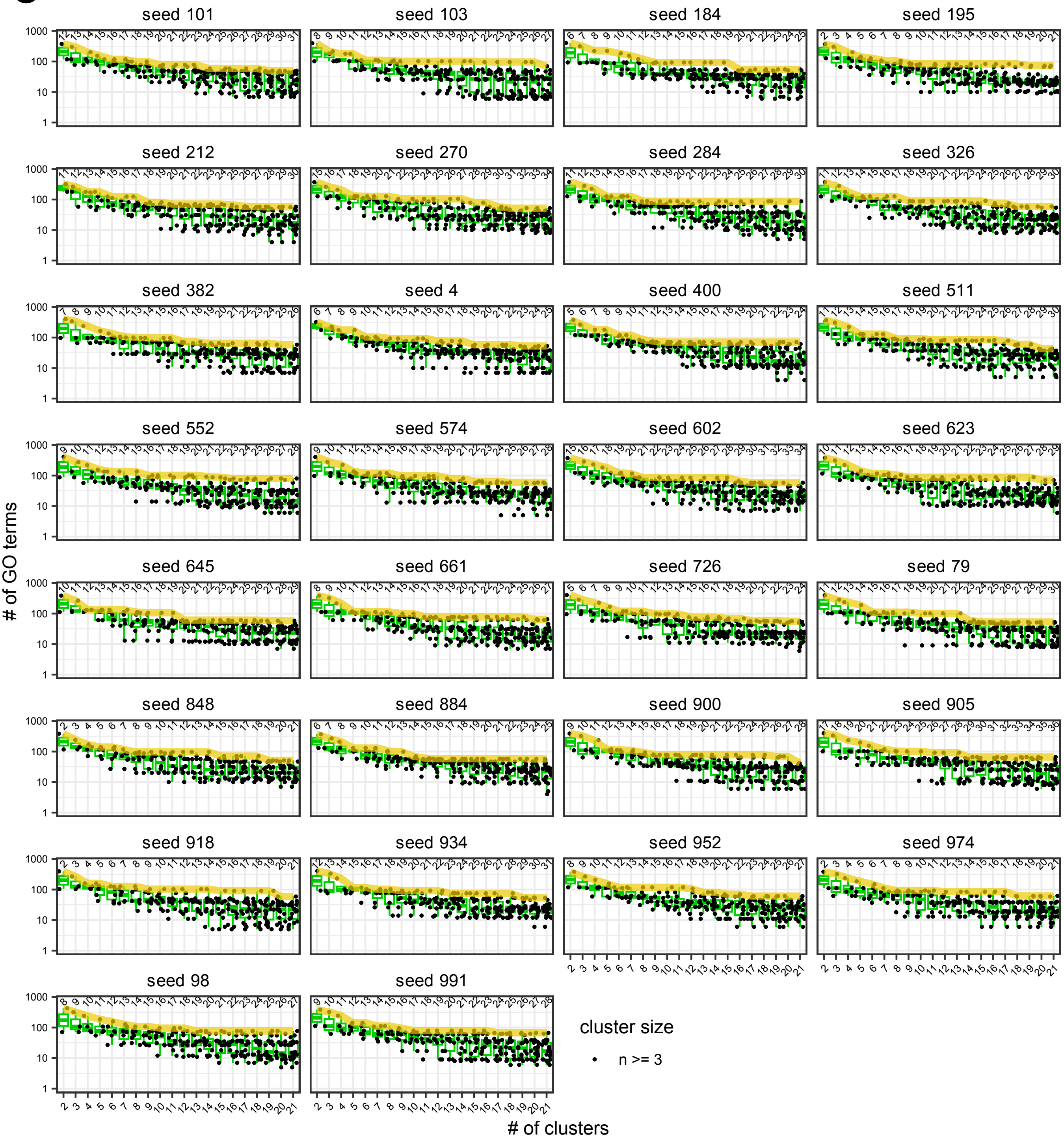

seed 101
seed 103
seed 184
seed 195
seed 212
seed 270
seed 284
seed 326
seed 382
seed 4
seed 400
seed 511
seed 552
seed 574
seed 602
seed 623
seed 645
seed 661
seed 726
seed 79
seed 848
seed 884
seed 900
seed 905
seed 918
seed 934
seed 952
seed 974
seed 98
seed 991
# of GO terms
# of clusters
cluster size
n >= 3

D

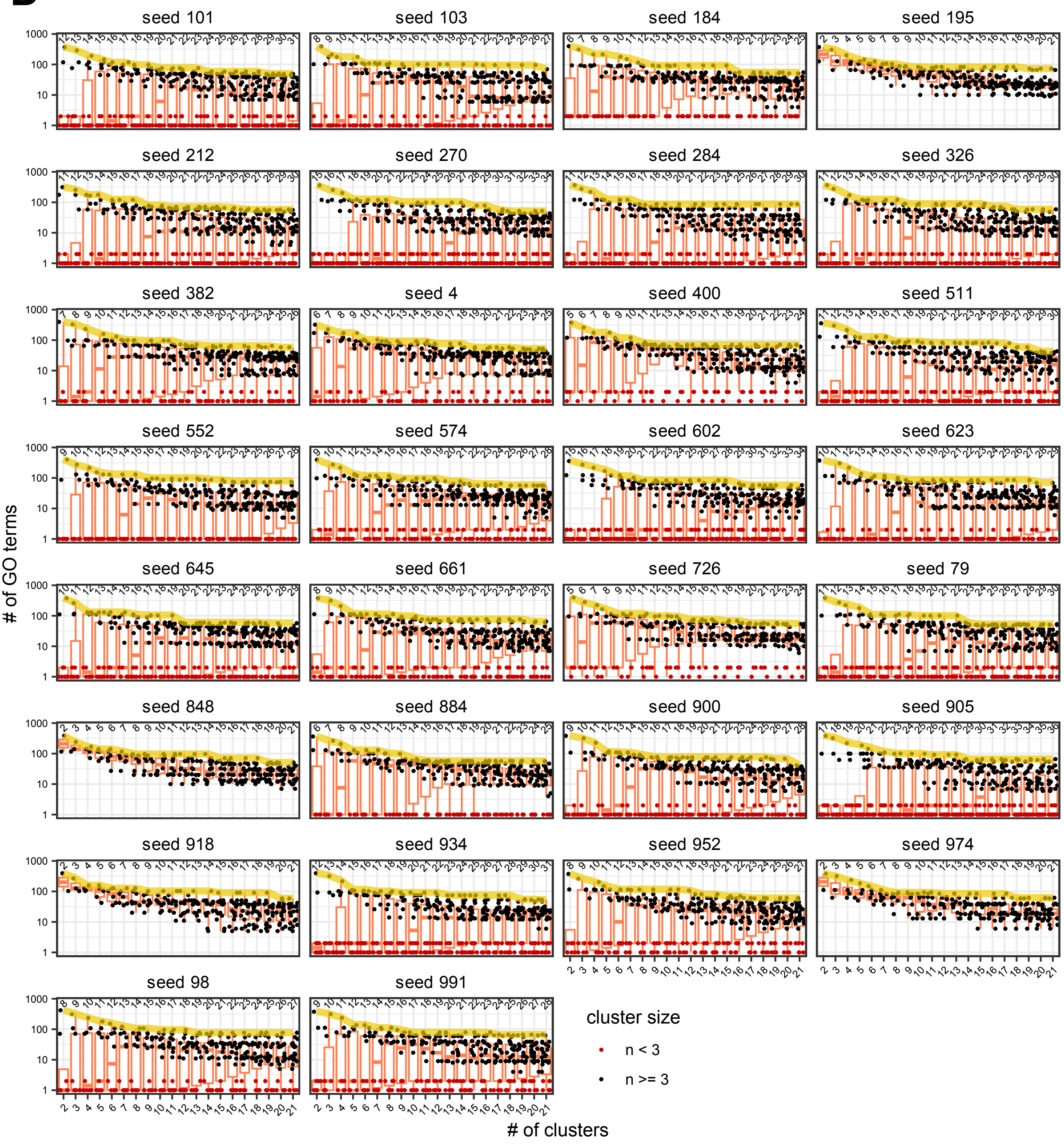
seed 101
seed 103
seed 184
seed 195
seed 212
seed 270
seed 284
seed 326
seed 382
seed 4
seed 400
seed 511
seed 552
seed 574
seed 602
seed 623
seed 645
seed 661
seed 726
seed 79
seed 848
seed 884
seed 900
seed 905
seed 918
seed 934
seed 952
seed 974
seed 98
seed 991
# of GO terms
# of clusters
cluster size
n < 3
n >= 3

E

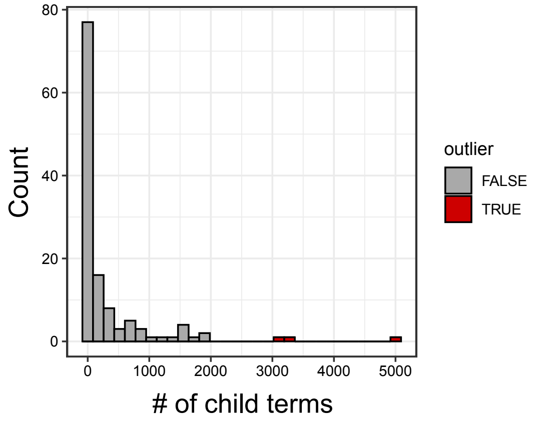

Count
80
60
40
20
0
0
1000
2000
3000
4000
5000
# of child terms
outlier
FALSE
TRUE

Fig. S3

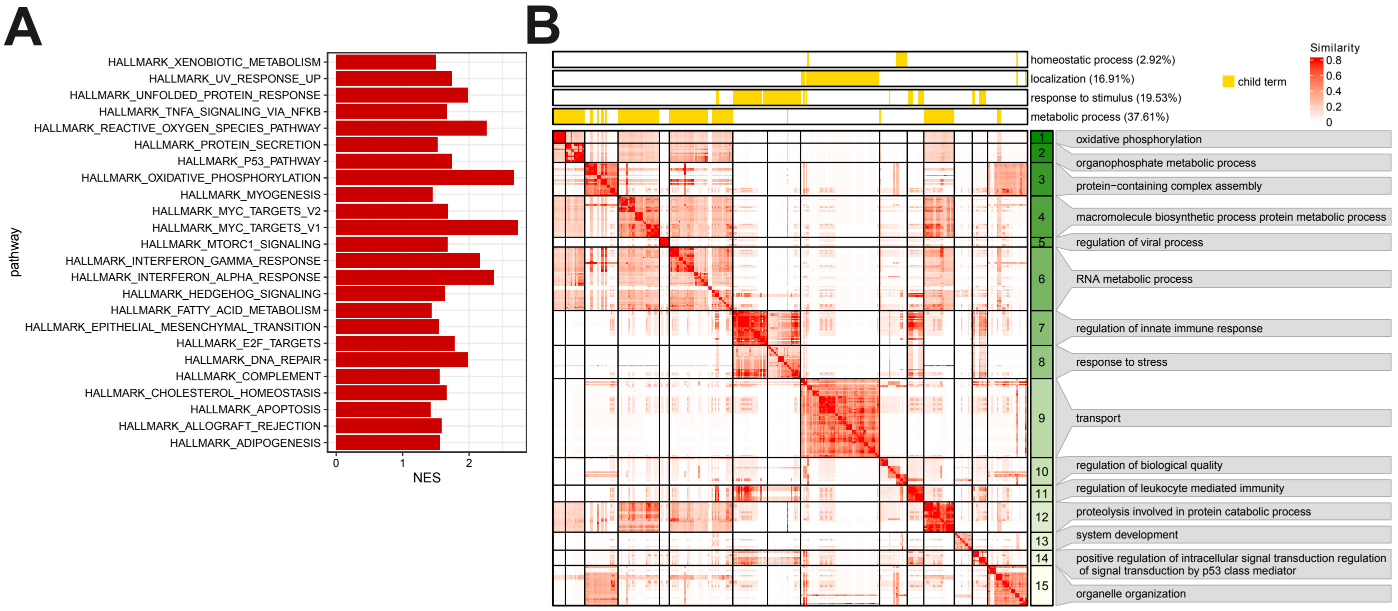
A
HALLMARK_XENOBIOTIC_METABOLISM
HALLMARK_UV_RESPONSE_UP
HALLMARK_UNFOLDED_PROTEIN_RESPONSE
HALLMARK_TNFA_SIGNALING_VIA_NFKB
HALLMARK_REACTIVE_OXYGEN_SPECIES_PATHWAY
HALLMARK_PROTEIN_SECRETION
HALLMARK_P53_PATHWAY
HALLMARK_OXIDATIVE_PHOSPHORYLATION
HALLMARK_MYOGENESIS
HALLMARK_MYC_TARGETS_V2
HALLMARK_MYC_TARGETS_V1
HALLMARK_MTORC1_SIGNALING
HALLMARK_INTERFERON_GAMMA_RESPONSE
HALLMARK_INTERFERON_ALPHA_RESPONSE
HALLMARK_HEDGEHOG_SIGNALING
HALLMARK_FATTY_ACID_METABOLISM
HALLMARK_EPITHELIAL_MESENCHYMAL_TRANSITION
HALLMARK_E2F_TARGETS
HALLMARK_DNA_REPAIR
HALLMARK_COMPLEMENT
HALLMARK_CHOLESTEROL_HOMEOSTASIS
HALLMARK_APOPTOSIS
HALLMARK_ALLOGRAFT_REJECTION
HALLMARK_ADIPOGENESIS
pathway
0
1
2
NES
B
homeostatic process (2.92%)
localization (16.91%)
response to stimulus (19.53%)
metabolic process (37.61%)
child term
Similarity
0.8
0.6
0.4
0.2
0
1 oxidative phosphorylation
2 organophosphate metabolic process
3 protein−containing complex assembly
4 macromolecule biosynthetic process protein metabolic process
5 regulation of viral process
6 RNA metabolic process
7 regulation of innate immune response
8 response to stress
9 transport
10 regulation of biological quality
11 regulation of leukocyte mediated immunity
12 proteolysis involved in protein catabolic process
13 system development
14 positive regulation of intracellular signal transduction regulation of signal transduction by p53 class mediator
15 organelle organization

C

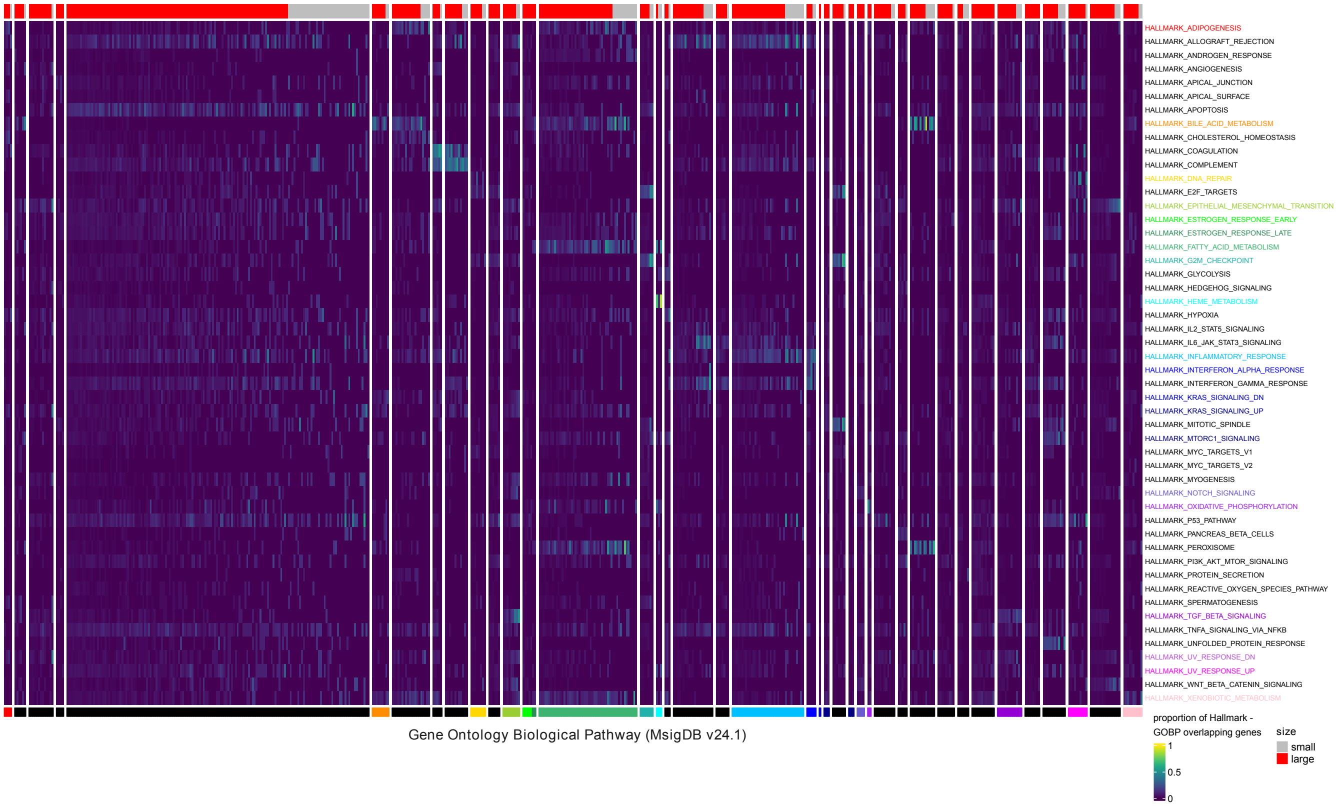

HALLMARK_ADIPOGENESIS
HALLMARK_ALLOGRAFT_REJECTION
HALLMARK_ANDROGEN_RESPONSE
HALLMARK_ANGIOGENESIS
HALLMARK_APICAL_JUNCTION
HALLMARK_APICAL_SURFACE
HALLMARK_APOPTOSIS
HALLMARK_BILE_ACID_METABOLISM
HALLMARK_CHOLESTEROL_HOMEOSTASIS
HALLMARK_COAGULATION
HALLMARK_COMPLEMENT
HALLMARK_DNA_REPAIR
HALLMARK_E2F_TARGETS
HALLMARK_EPITHELIAL_MESENCHYMAL_TRANSITION
HALLMARK_ESTROGEN_RESPONSE_EARLY
HALLMARK_ESTROGEN_RESPONSE_LATE
HALLMARK_FATTY_ACID_METABOLISM
HALLMARK_G2M_CHECKPOINT
HALLMARK_GLYCOLYSIS
HALLMARK_HEDGEHOG_SIGNALING
HALLMARK_HEME_METABOLISM
HALLMARK_HYPOXIA
HALLMARK_IL2_STAT5_SIGNALING
HALLMARK_IL6_JAK_STAT3_SIGNALING
HALLMARK_INFLAMMATORY_RESPONSE
HALLMARK_INTERFERON_ALPHA_RESPONSE
HALLMARK_INTERFERON_GAMMA_RESPONSE
HALLMARK_KRAS_SIGNALING_DN
HALLMARK_KRAS_SIGNALING_UP
HALLMARK_MITOTIC_SPINDLE
HALLMARK_MTORC1_SIGNALING
HALLMARK_MYC_TARGETS_V1
HALLMARK_MYC_TARGETS_V2
HALLMARK_MYOGENESIS
HALLMARK_NOTCH_SIGNALING
HALLMARK_OXIDATIVE_PHOSPHORYLATION
HALLMARK_P53_PATHWAY
HALLMARK_PANCREAS_BETA_CELLS
HALLMARK_PEROXISOME
HALLMARK_PI3K_AKT_MTOR_SIGNALING
HALLMARK_PROTEIN_SECRETION
HALLMARK_REACTIVE_OXYGEN_SPECIES_PATHWAY
HALLMARK_SPERMATOGENESIS
HALLMARK_TGF_BETA_SIGNALING
HALLMARK_TNFA_SIGNALING_VIA_NFKB
HALLMARK_UNFOLDED_PROTEIN_RESPONSE
HALLMARK_UV_RESPONSE_DN
HALLMARK_UV_RESPONSE_UP
HALLMARK_WNT_BETA_CATENIN_SIGNALING
HALLMARK_XENOBIOTIC_METABOLISM
Gene Ontology Biological Pathway (MsigDB v24.1)
proportion of Hallmark - GOBP overlapping genes
1
0.5
0
size
small
large

D

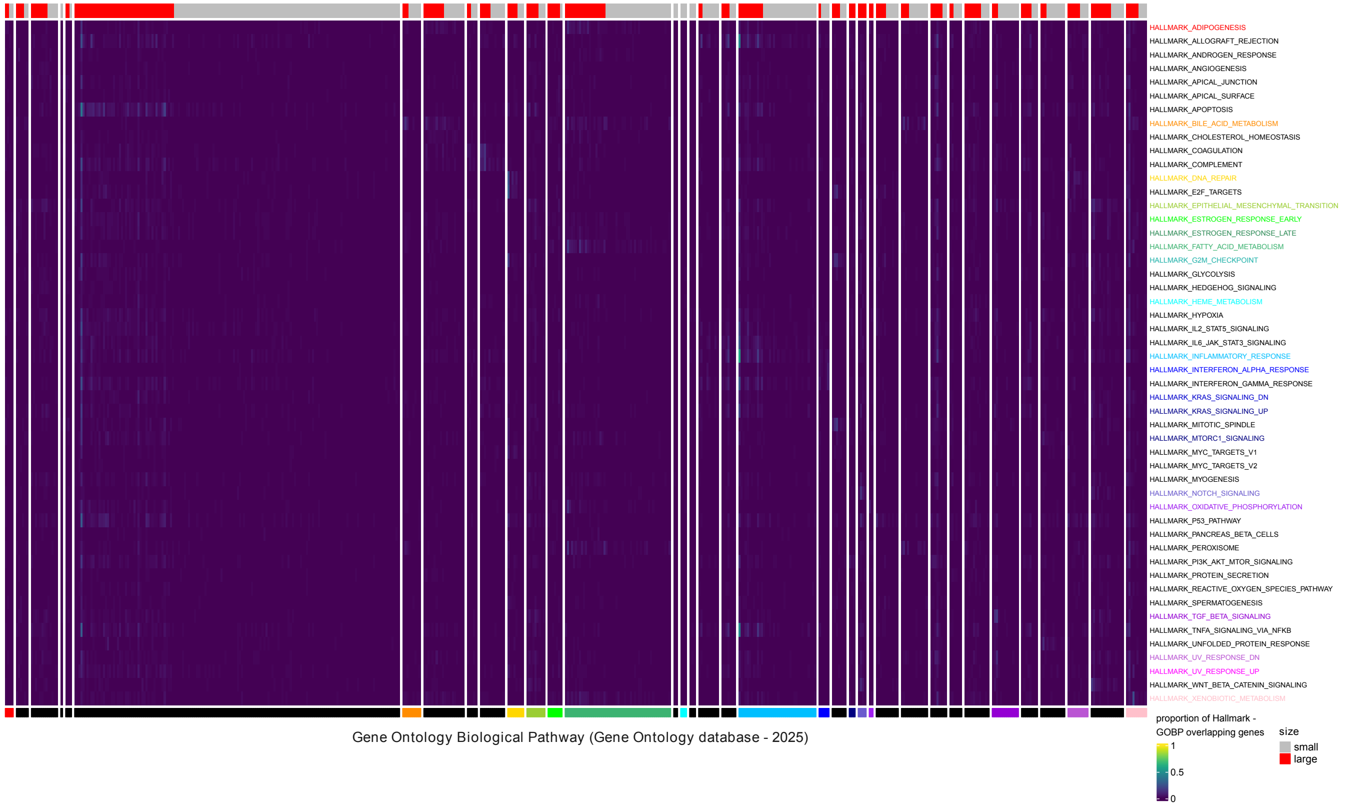

HALLMARK_ADIPOGENESIS
HALLMARK_ALLOGRAFT_REJECTION
HALLMARK_ANDROGEN_RESPONSE
HALLMARK_ANGIOGENESIS
HALLMARK_APICAL_JUNCTION
HALLMARK_APICAL_SURFACE
HALLMARK_APOPTOSIS
HALLMARK_BILE_ACID_METABOLISM
HALLMARK_CHOLESTEROL_HOMEOSTASIS
HALLMARK_COAGULATION
HALLMARK_COMPLEMENT
HALLMARK_DNA_REPAIR
HALLMARK_E2F_TARGETS
HALLMARK_EPITHELIAL_MESENCHYMAL_TRANSITION
HALLMARK_ESTROGEN_RESPONSE_EARLY
HALLMARK_ESTROGEN_RESPONSE_LATE
HALLMARK_FATTY_ACID_METABOLISM
HALLMARK_G2M_CHECKPOINT
HALLMARK_GLYCOLYSIS
HALLMARK_HEDGEHOG_SIGNALING
HALLMARK_HEME_METABOLISM
HALLMARK_HYPOXIA
HALLMARK_IL2_STAT5_SIGNALING
HALLMARK_IL6_JAK_STAT3_SIGNALING
HALLMARK_INFLAMMATORY_RESPONSE
HALLMARK_INTERFERON_ALPHA_RESPONSE
HALLMARK_INTERFERON_GAMMA_RESPONSE
HALLMARK_KRAS_SIGNALING_DN
HALLMARK_KRAS_SIGNALING_UP
HALLMARK_MITOTIC_SPINDLE
HALLMARK_MTORC1_SIGNALING
HALLMARK_MYC_TARGETS_V1
HALLMARK_MYC_TARGETS_V2
HALLMARK_MYOGENESIS
HALLMARK_NOTCH_SIGNALING
HALLMARK_OXIDATIVE_PHOSPHORYLATION
HALLMARK_P53_PATHWAY
HALLMARK_PANCREAS_BETA_CELLS
HALLMARK_PEROXISOME
HALLMARK_PI3K_AKT_MTOR_SIGNALING
HALLMARK_PROTEIN_SECRETION
HALLMARK_REACTIVE_OXYGEN_SPECIES_PATHWAY
HALLMARK_SPERMATOGENESIS
HALLMARK_TGF_BETA_SIGNALING
HALLMARK_TNFA_SIGNALING_VIA_NFKB
HALLMARK_UNFOLDED_PROTEIN_RESPONSE
HALLMARK_UV_RESPONSE_DN
HALLMARK_UV_RESPONSE_UP
HALLMARK_WNT_BETA_CATENIN_SIGNALING
HALLMARK_XENOBIOTIC_METABOLISM
Gene Ontology Biological Pathway (Gene Ontology database - 2025)
proportion of Hallmark - GOBP overlapping genes
1
0.5
0
size
small
large
