# Supplementary material for: GOREA: Unbiased Interpretation of Functional Enrichment
Source: Mol Cells. 2025 Sep 24;48(11):100283. doi: 10.1016/j.mocell.2025.100283 (PMC12552962; doi:10.1016/j.mocell.2025.100283)
Supplement: Supplementary file 3 — Legned for Supplementary Figures [file mmc3.pdf]

Fig. S1. GOBP terms reported in the paper among all significant terms. (A), (C), (E) Bar plots showing significant GOBP terms from each paper. Red bars indicate reported GOBP in the paper's figures. (B) Total number of GOBP terms from (A). (D) Total number of GOBP terms from (C). (F) Total number of GOBP terms from (E). (B), (D), (F) Red regions refer to the number of reported GOBP in the paper's figure, among significant GOBP terms.

Fig. S2. Comparison of three clustering methods using 500 GOBP terms. (A) Box plots indicating size of clusters from binary cut and hierarchical clustering method using 30 sets of 500 random GOBP terms. (B) Box plots and line plots indicating the results of binary cut for 30 sets of random 500 GOBP terms. (C) Box plots and line plots indicating the results of hierarchical clustering for 30 sets of 500 GOBPs. (D) Box plots and line plots indicating the results of combined clustering for 30 sets of 500 GOBPs. (B), (C), (D) Red dots indicate clusters with small size, and black dots mean clusters including 3 or more GOBP terms. Yellow lines indicate the largest cluster in each box plot. Additionally, the numbers indicated in the top of box plot are total number of clusters in each cutoff. (E) Histogram showing child GOBP terms with level 1 and 2.

Fig. S3. Comparison between GOBP and cancer related gene sets. (A) Bar plot for the GSEA result using Hallmark. (B) GOREA result for the GSEA result using GOBP. (C) Heatmap showing proportion of overlapping genes between Hallmark and the latest GOBP (v24.1) from MsigDB. (D) Heatmap showing proportion of overlapping genes between Hallmark and GOBP from GeneOntology website. (C), (D) "Large" and "Small" refer to the size of the gene sets. "Large" indicates GOBP terms containing more than 10 genes, while "Small" refers to those with 10 or fewer genes. The colors shown in the Hallmark and bottom GOBP annotations represent the presence of Hallmark-related keywords within the GOBP terms.
